# Successful management of perivascular epithelioid cell tumor of the rectum with recurrent liver metastases: A case report

**DOI:** 10.1097/MD.0000000000011679

**Published:** 2018-08-03

**Authors:** Kung-Hung Lin, Nai-Jen Chang, Li-Ren Liou, Ming-Shan Su, Min-Jen Tsao, Meng-Lin Huang

**Affiliations:** a Department of Surgery, Division of General Surgery; b Division of Pathology, Zuoying Branch of Kaohsiung Armed Forces General Hospital, Taiwan, Republic of China.

**Keywords:** HMB45, PEComa, perivascular epithelioid cell tumor, rectum

## Abstract

**Rationale:**

The perivascular epithelioid cell tumor (PEComa) is rare in young man and rarely occurs in the large intestine.

**Patient concerns:**

The clinical characteristics, diagnosis, and managements in a 28-year-old boy who presented with sudden onset of cramping and abdominal pain and intermittent melena with a blood pressure of 74/39 mm Hg was retrospectively reviewed. CT scan of the abdomen revealed a 8.9 × 7.2 cm mass in the pelvic floor.

**Diagnoses:**

Given the difficulty of obtaining a diagnostic specimen, surgical resection was performed. The pathology report of lower anterior resection was malignant PEComa of the rectum in 2006.

**Interventions:**

Treatment consisted of surgical resection only without additional adjuvant therapy. Over the next 49 months (until 2010) after surgery, abdominal CT showed a 0.6-cm hypodense mass over the liver with suspected liver metastasis. He refused any further evaluation and treatment. After 4 years (2014), abdominal CT showed that the original mass had increased from 0.6 to 1.5 cm and the number of tumors had increased from 1 to 3. In August 2014, he underwent a metastatic hepatectomy without additional chemotherapy or radiotherapy.

**Outcomes:**

We noted that the metastatic progression was slow in the 4 years after the first operation. At 28 months after metastatic hepatectomy, the patient was doing well. There was also no recurrence of the PEComa of the rectum at the 120-month follow-up in 2016.

**Lessons:**

To the best of our knowledge, this is the first report of a PEComa of the rectum with liver metastases treated with only surgical resection. At approximately 8.8 cm, this is the largest PEComa of the rectum reported in the recent literature.

## 1 Introduction

The perivascular epithelioid cell (PEC) is a cell type that is histologically and immunohistochemically present in a group of tumors, including renal angiomyolipoma (AML), clear-cell sugar tumor (CCST) lymphangioleiomyomatosis (LAM), and rare clear-cell tumors of other anatomical sites.^[1]^ In 1963, Liebow and Castleman^[2]^ first identified a distinct type of benign clear cell tumor referred to as CCST in the lung. The same authors coined the term “benign clear cell (sugar) tumor” of the lung in 1971.^[3]^ The name refers to the clear cytoplasm of the cells, which is rich in glycogen. The cell of origin and different types of clear cell tumors of the lung (the so-called sugar tumor) have been enigmatic and controversial for the last 3 decades. Zamboni et al^[4]^ asserted that clear cell tumors of the lung arise from the “perivascular epithelioid cell” (PEC) and noted that similar cells have been identified in AML and lymphangiomyomas. Tazelaar et al^[5]^ reported a case described as primary extrapulmonary sugar tumors (PESTs) of the vulva. The modifier “primary extrapulmonary” was used to emphasize that these tumors are not restricted to the lung.

There have been increasing numbers of reports of different views on and insights into clear cell tumors in recent years. The existence of PECs was first reported by Bonetti et al^[6]^ in 1992. The term PEComa was introduced by Zamboni et al^[4]^ in 1996 to describe this rare family of morphological, immunohistochemical, and ultrastructural features. The World Health Organization defines PEComa as unusual mesenchymal tumors composed of histologically and immunohistochemically distinctive PECs.^[7]^ Currently, PEComa is a widely known and accepted entity. We present a case of rectum PEComa in a young male who initially presented with an area of intratumoral hemorrhage.

## 2 Case report

A 28-year-old male was transferred to our hospital in November 2006 because of a sudden onset of cramping and abdominal pain and intermittent melena for 4 days. Initially, he exhibited symptoms including generalized weakness, dizziness, and massive bloody stool passage. There was no significant past medical history of recent infection, inflammatory bowel disease, bleeding disorders, changes in bowel habits, significant weight loss, or tuberous sclerosis complex. The patient had no previous surgeries, and the family history was unremarkable. The patient's height was 180.2 cm, weight was 78.5 kg, blood pressure was 74/39 mm Hg, pulse rate was 122 beats/min, and temperature was 36.4°C. The physical examination revealed moderate distention and tenderness in the left lower quadrant with associated defense. On digital rectal examination, there was some gross blood mixed with soft stool and an empty ampulla. Laboratory data showed a white blood cell count of 17,120/mm^3^, hemoglobin level of 7.4 g/dL, hematocrit of 23.9%, and platelet count of 162,000/mm^3^. Other biochemical tests were normal. The serum carcinoembryonic antigen level was normal.

**Figure 1:**
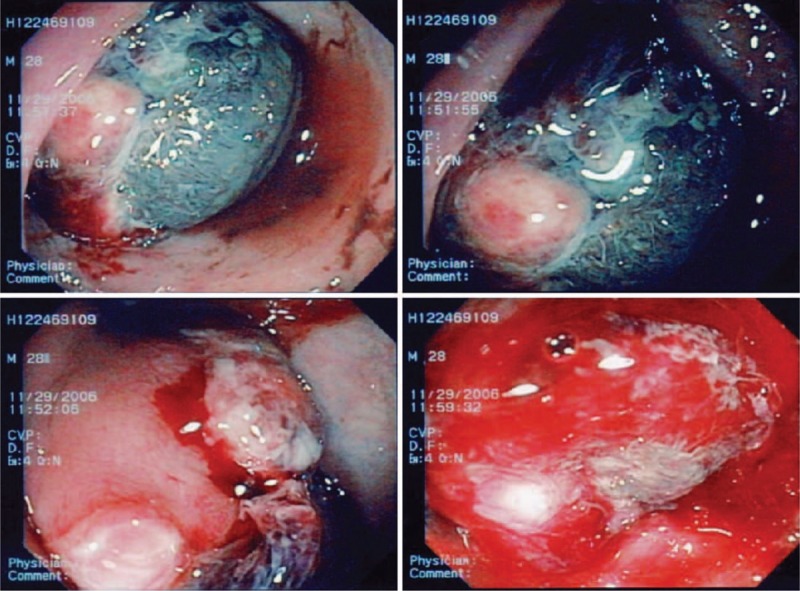
The colonoscopy revealed a large rectal tumor with easy bleeding 15 cm from the anal verge.

**Figure 2:**
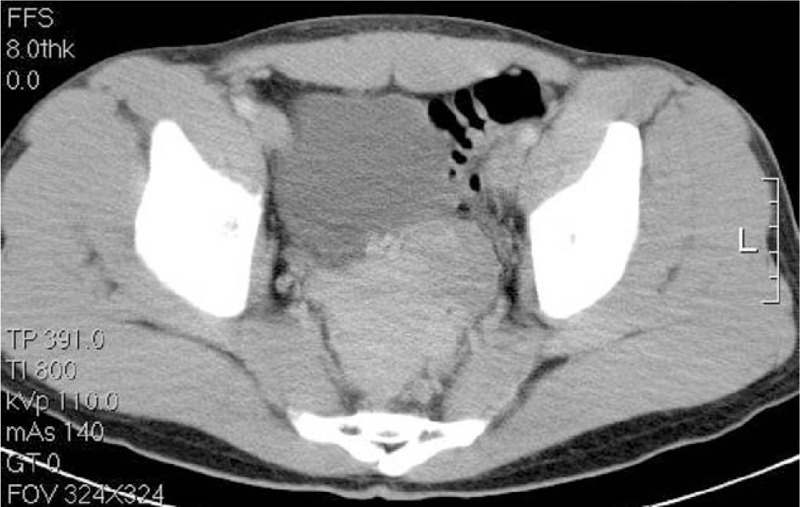
Abdominal computed tomography scan, horizontal view, revealing an 8.9 × 7.2 cm heterogeneous mass with a low-density area and wall thickening, suggesting a gastrointestinal stromal tumor.

The gastroscopic evaluation was normal. The full colonoscopy revealed a greater than 4.0 cm in diameter, large ulceration with an easy bleeding mass of the rectum lying beneath the mucosa but protruding into the lumen 15 cm from the anal verge (Fig. 1). This tumor could not be classified by biopsy. However, on the basis of the immunohistopathological features, carcinoma and malignant lymphoma could be excluded. An abdominal enhanced CT scan revealed a heterogeneous mass lesion of approximately 8.9 x 7.2 cm in the pelvic floor at approximately the level of the rectum (Fig. 2). A technetium-99m red blood cell scan showed no significant findings. Repeated attempts at endoscopic revaluation resulted in profuse bleeding requiring blood transfusions. A diagnostic biopsy failed before the surgical treatment due to bleeding from a light touch of the mass lesion

**Figure 3:**
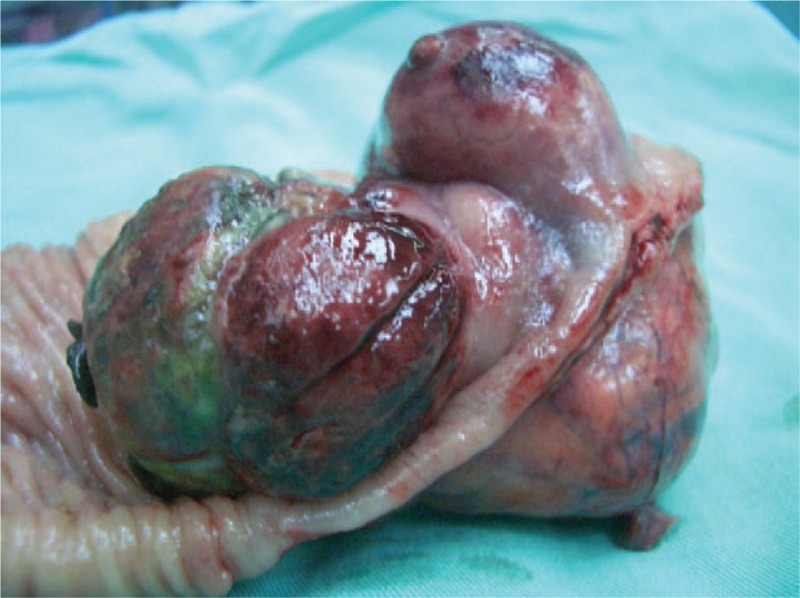
A large green to dark-red mass with gross mucosa rupture and submucosa protruding into the lumen.

**Figure 4:**
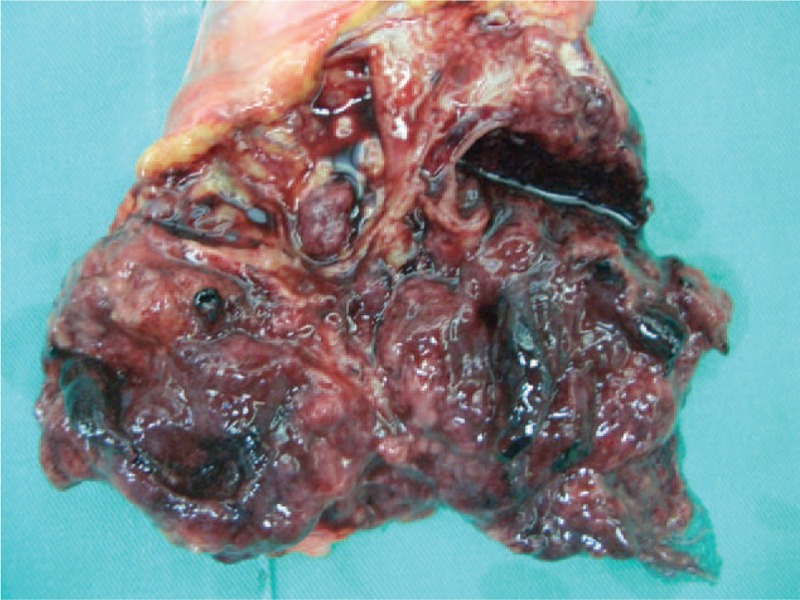
The tumor showed dark red-grey solid parenchyma and irregular cystic spaces with colorless serous liquid.

**Figure 5:**
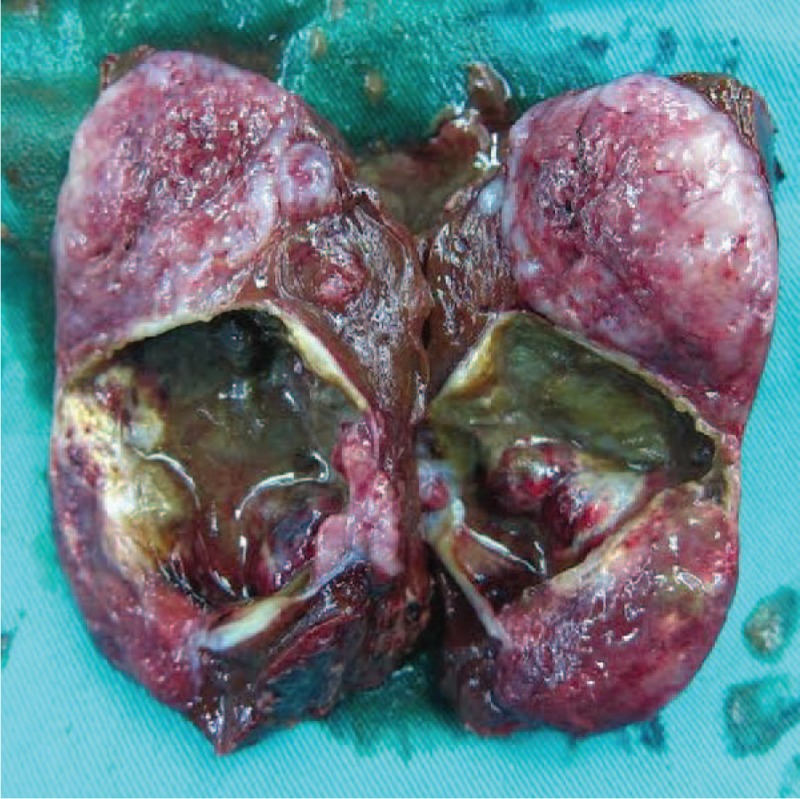
The cut surface showed a yellowish-tan to gray-red solid parenchyma with focal irregular cystic spaces containing colorless serous fluid.

**Figure 6:**
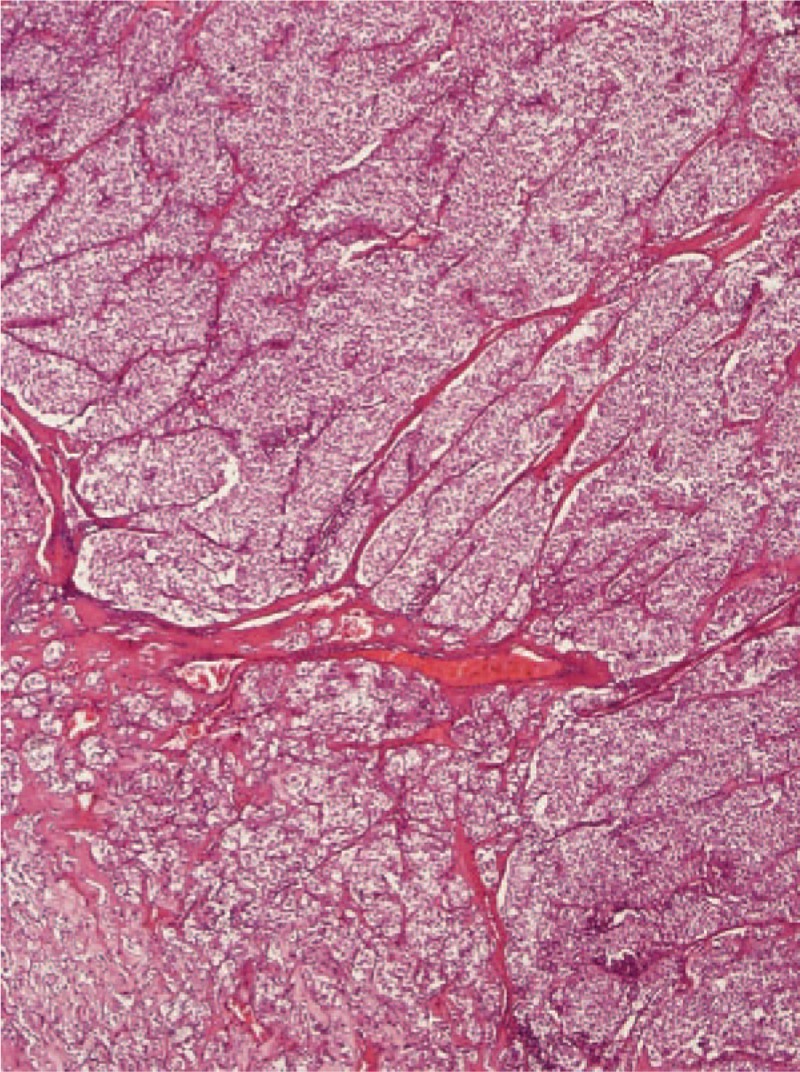
(40 × magnification) Low-power view of protruding tumor composed of nests, trabeculae, or sheets of epithelioid cells with thin-walled vessels.

**Figure 7:**
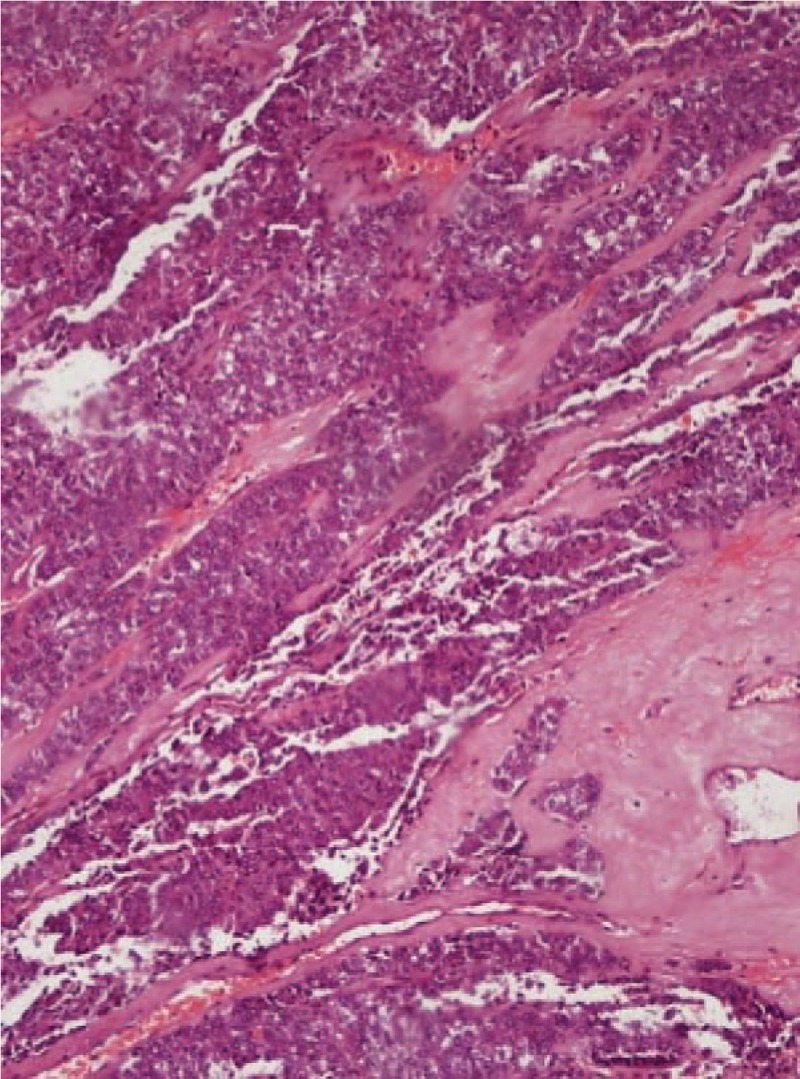
(100 × magnification) Perivascular hyalinization and spindle cells around small blood vessels are noted.

**Figure 8:**
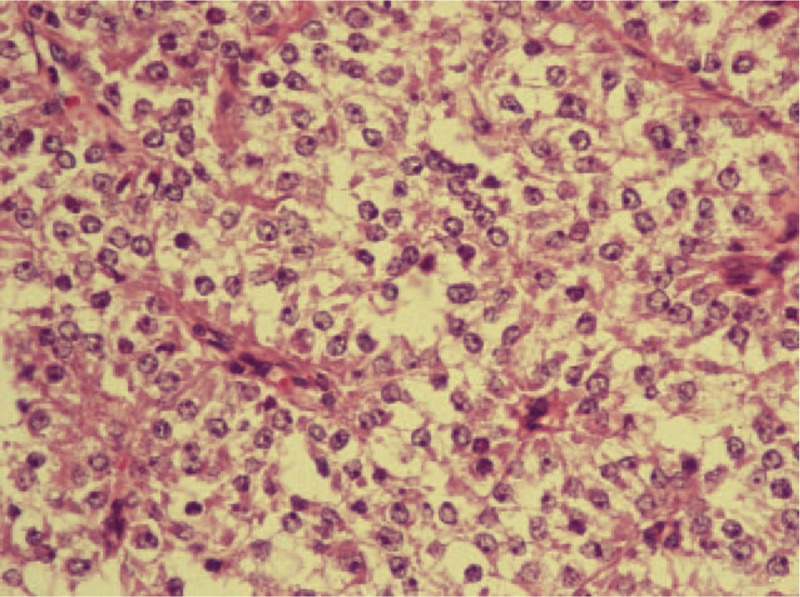
(400 × magnification) High-power view of tumor cells shows clear to granular, light eosinophilic cytoplasm and round to oval nuclei with distinct small nucleoli.

**Figure 9:**
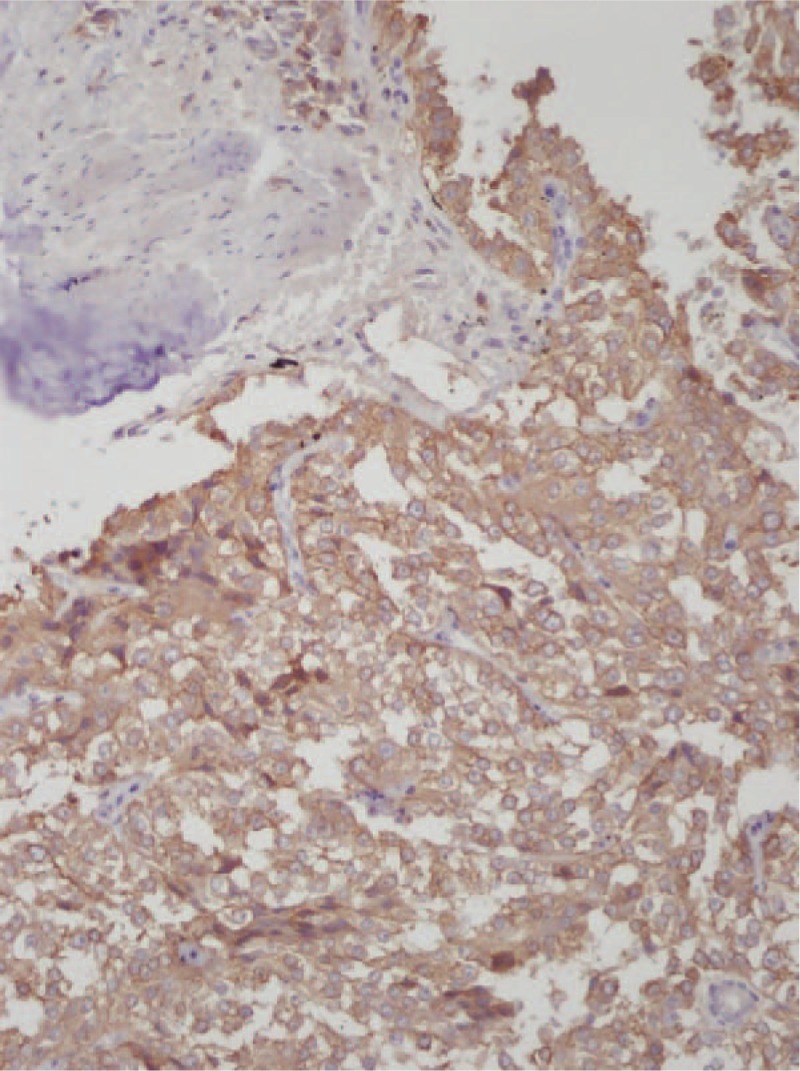
(400 × magnification) Immunohistochemical staining revealed positive findings for HMB-45.

Given the difficulty of obtaining a diagnostic specimen, surgical resection and lower anterior resection were performed. On gross examination, the resected specimen was an 8.8 x 5.5 x 4.5 cm, tan, gray-white, soft, and well-circumscribed transmural mass of the rectum, mainly involving the muscularis propria and protruding into the tunica adventitia. The mucosa and submucosa were intact (Fig. 3). The tumor protruded into the lumen, and the overlying mucosa showed ulceration. The tumor had a dark, red-grey, solid parenchyma with irregular cystic spaces with colorless serous liquid (Fig. 4). The cut surface showed a yellowish-tan to gray-red solid parenchyma with focal irregular cystic spaces containing colorless serous fluid (Fig. 5). All surgical margins were macroscopically free of tumor. No separate polyps were identified. Microscopically, the foci of hemorrhage and necrosis were present. The tumor extended through the muscularis propria into the subserosa tissue with lymphatic invasion. The colon mucosal tissue was composed of sheets with atypical glands with spindle-to-epithelioid cells and nuclear abnormalities in a tubular arrangement (Fig. 6). Perivascular hyalinization was noted (Fig. 7). Most tumor nuclei showed clear to granular, light, eosinophilic cytoplasm, and round to oval nuclei with distant small nucleoli pleomorphism (Fig. 8). Less than 50% of the tumor area was necrotic. The mitotic rate was low. One of 27 accompanying serosal lymph nodes contained metastatic tumors that distended the subcapsular sinus. All of the surgical margins were free of tumor. Immunohistochemically, the tumor cells were positive for melanoma-associated antigen (HMB-45) (Fig. 9) but negative for cytokeratin, c-kit, synaptophysin, S-100, and actin. A diagnosis of metastatic PEComa was made after examination of the resection material.

Unfortunately, the patient did not complete the follow-up in the outpatient department after surgery because he relocated to another city to seek work. At the 49th month (2010) postoperatively, the patient returned to the clinic upon our scheduled request. An abdominal CT scan showed a 0.6 cm hypodense mass over the liver. He refused further evaluation and treatment. In July 2014, during the course of a required physical health check, he received an abdominal CT that showed that the original mass had increased from 0.6 to 1.5 cm and that the number of tumors had increased from 1 to 3 compared with the previous CT image in 2010. We noted that the distant liver metastasis progressed very slowly during the 4 years of follow-up. The liver function and the other serum tumor markers were normal upon examination. In August 2014, he underwent segmental hepatectomy (S3, S4A, S5, and S6) combined with wedge resection. The pathology report of segmental hepatectomy was metastatic PEComa.

This patient underwent 2 different surgical resections at 2 different times. He underwent the lower anterior resection of the PEComa of the rectum in 2006 and received the segmental hepatectomy in 2014. He is currently undergoing regular surveillance and has remained free of disease 28 months after the second operation. At the follow-up examination, the patient felt well, and the general clinical examination, subsequent colonoscopies, and abdominal CT scan once every 3 months revealed no significant findings after second operation in 2014. Since the primary surgery in 2006, there was also no recurrence of the PEComa of the rectum according to the general clinical examination, subsequent colonoscopies, and abdominal CT scan at the 120-month follow-up of the very first instance back in 2006.

## 3 Discussion

PEComa was introduced to describe a family of tumors, including AML, pulmonary and extrapulmonary CCST, LAM, and similar lesions arising at a variety of visceral and soft tissue sites, all of which are characterized by the same morphological, immunohistochemical, and ultrastructural features.^[4,8,9]^ This rare neoplasm seems to arise most commonly at retroperitoneal, visceral, abdominal, and pelvic sites but has been reported at almost every body site, and the growing list of reported sites include gastrointestinal, gynecologic, and genitourinary sites, the extremities, somatic soft tissue, and the skin; among these sites are the falciform ligament, uterus, uterine cervix, liver, kidney, lung, breast, cardiac septum, pancreas, prostate, thigh, and gastrointestinal tract.^[1,9–13]^ These tumors all share a distinctive cell type, the PEC, which has no known normal tissue counterpart.^[8]^

We reviewed the literature on gastrointestinal tract PEComa over the past 20 years, finding that malignant PEComa of the colon is extremely rare. Given the relative rarity of this group of tumors and that it was not delineated until the mid-1990s, it has not been possible to fully define criteria for malignancy in PEComas. The clinicopathological features of a total of 32 sporadic cases are summarized in Tables 1 and 2. The ratio of males to females was 12 to 20, suggesting that primary PEComa of the colon is more common in females. The incidence was markedly higher in adults (62.5%) than in children (37.5%). The mean age at the diagnosis of primary PEComa of the colon was 29.9 years (range 5–62 years). The mean diameter of the primary PEComa of the colon was 46.73 mm (range 12–120 mm). The most common site of the lesion was the rectum (n = 9), follow by the sigmoid colon (n = 7), the cecum (n = 6), the ascending colon (n = 4), the descending colon (n = 3), the transverse colon (n = 2), and the appendix (n = 1). The most common sites of lesion metastases were the liver (n = 2), peritoneum (n = 1), and pancreas (n = 1). In our patient, the tumor was classified as malignant because the tumor size was 8.8 cm (larger than 5 cm), the tumor had a high nuclear grade and an infiltrative growth pattern, and 1 of 27 accompanying lymph nodes contained metastatic tumors (aggressive clinical behavior). In addition, tumors of uncertain malignant potential had either nuclear pleomorphism and multinucleated giant cells only or a size larger than 5 cm. Tumors with 2 or more worrisome features were classified as malignant. Overall, close to 83% (20/24) of patients were alive with no evidence of disease at follow-up, excluding primary metastasis (Table 1). Surgical resection was performed in all patients, and only 3 received adjuvant chemotherapy. One 15-year-old girl presented with polypoid PEComa in the rectum and was treated by surgical resection and adjuvant chemotherapy. She was doing well at the 9-month follow-up, with neither radiologic nor endoscopic evidence of recurrence.^[12]^ A 7-year-old boy presented with a 3.7-cm PEComa in the ascending colon and first underwent adjuvant interferon-α 2b therapy.^[14]^ The other patient was a 23-year-old male who received adjuvant chemotherapy with doxorubicin and ifosfamide.^[15]^ However, the latter patient had evidence of local recurrence and liver metastasis at 4 months postoperatively and died of the tumor after 23 months.^[15]^

**Table 1:**
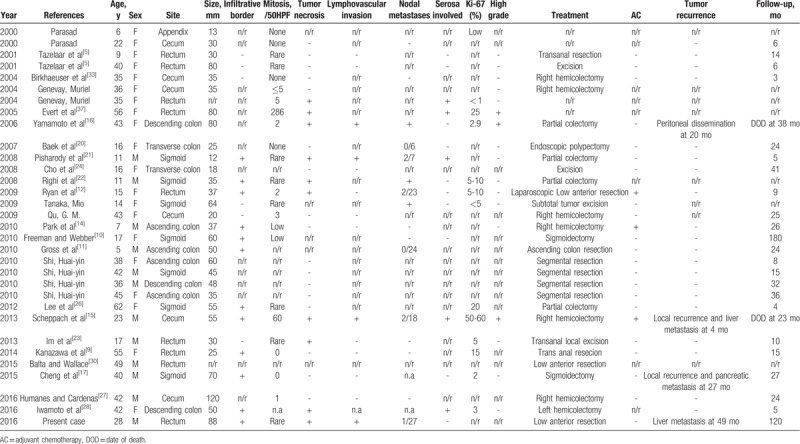
Patient characteristics and tumor morphologic and histopathological features and outcomes.

**Table 2:**
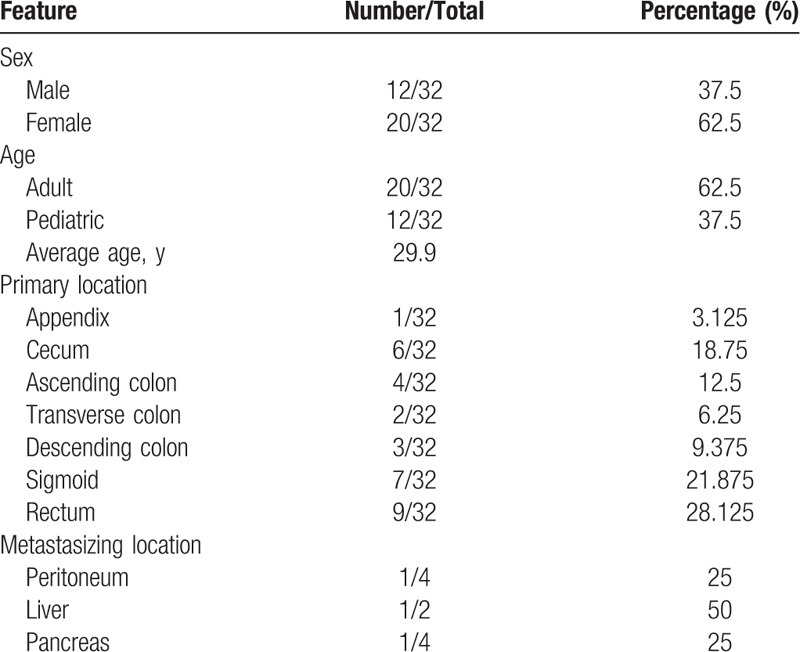
Clinicopathologic features of gastrointestinal perivascular epithelioid cell tumors-not otherwise specified.

Follow-up data were available for only 25 of the 32 patients, and the median follow-up duration was 28.8 months (range 3–180 months) after surgery. Four cases, including our presented case, had recurrence,^[15–17]^ 3 of whom underwent re-resection of their tumors. The 2-year disease-free survival rate of the 12 patients was 91.7% (11/12). Two patients died of their disease. One patient died 38 months after the first operation, and the other patient died 23 months after the first operation.

### 3.1 Clinical presentation

PEComas can arise in patients of almost any age, but the precise incidence of gastrointestinal PEComas is not known. In 1999, Domoto et al^[18]^ reported that the incidence of colon neoplasms was less than 0.1%. Folpe et al^[19]^ asserted that the peak incidence was in the fourth decade of life. In addition, there was a marked female preponderance.^[9,12,13]^ In 2016, Chen et al^[13]^ reported in a review study of gastrointestinal PEComa that the gastrointestinal tract is the second most frequent site of PEComa, with the most frequent site being the gynecological tract. They further indicated that gastrointestinal PEComas of the colon and rectum were most common.^[13]^ In addition, there were several cases of PEComa in the gastrointestinal tract that were reported in both the pediatric^[10,11,14,20–25]^ and adult populations^[9,15,17,26–30]^ over the last 2 decades. In 2000, Prasad et al^[25]^ reported the first child with PEComa involving the appendix. In 2008, Pisharody et al^[21]^ reported the first child with metastatic PEComa involving the sigmoid colon. In the study by Folpe et al,^[19]^ follow-up information on 26 patients (median follow-up period of 30 months) revealed local recurrence and metastases in 13% and 21% of patients, respectively. The most common sites of metastasis included the liver, lung, and bone.^[1]^ Until more cases of this extremely rare neoplasm are evaluated in a systemic review, definitive criteria for malignancy will remain uncertain.

### 3.2 Radiological diagnosis

Given the present situation, close follow-up, including imaging studies and colonoscopy, is mandatory after surgical resection of gastrointestinal PEComa, especially in patients with high-grade malignancy.^[9]^ Birkhaeuser et al^[31]^ suggest CT image evaluation at follow-up due to the unclear biological behavior of these tumors and the possibility of the rather aggressive behavior of these tumors at other locations. Most gastrointestinal PEComas manifest as a well-demarcated mass with homogeneous density in plain CT and show heterogeneous or homogenous enhancement in contrast-enhancement CT.^[13]^ The lesions are hypointense to isointense on T1-weighted imaging and heterogeneously hyperintense on T2-weighted imaging when applying magnetic resonance imaging (MRI).^[13]^ Ultrasonography may reveal a highly vascularized heterogeneous mass. However, abdominal CT, MRI, and ultrasonography are not sufficiently sensitive to enable the diagnosis of PEComas because of their nonspecific imaging characteristics.^[13]^

Freeman and Webber^[10]^ reported a case in which the longest period of follow-up for a colonic PEComa was 180 months. The patient, who underwent radical excision with a favorable outcome, was considered as a successful example of management.^[10]^ Pisharody et al^[21]^ presented a surveillance of a patient with physical examination and CT scans every 6 months, simultaneously, and a yearly endoscopy was also recommended. It is important that closed and long-term follow-up be accompanied by endoscopy and imaging for the purpose of ruling out local recurrence or distant metastasis of the tumor, especially in patients with high-grade malignancy.^[9]^ Close and long-term follow-up clinically and by CT scan is recommended. Endoscopy can help detect the lesions, which include a polypoid tumor or fungating mass protruding into the lumen with an ulcerated or smooth surface that has no specific sign.^[13]^

### 3.3 Pathological diagnosis

PEComa has been applied to an expanding family of tumors that are presumed to originate from microscopically and phenotypically unique PECs, although there is no known normal cellular counterpart to these cells.^[19]^ These tumors all share a distinctive cell type, the PEC, which has no known normal tissue counterpart.^[8]^

PEComas have morphologically, immunohistochemically, ultrastructurally, and genetically distinctive features such as an epithelioid appearance with a clear to granular cytoplasm, a round to oval, centrally located nucleus, and an inconspicuous nucleolus.^[1]^ The unifying concept proposed for this family of PEComas evolved over the past century and was developed by identifying unique clear cell features as well as the smooth muscle and melanocytic characteristics of the tumor cells. The surface of the neoplasm may be tan to gray and solid, firm, or even myxoid, and areas of hemorrhage or necrosis with a white-tan to gray-red color may be grossly observed. The overall cellularity is low to moderate, but some cases are highly cellular. PEComas are comprised of the following: plump, epithelioid cells with an abundant clear to lightly eosinophilic cytoplasm; round, medium-sized nuclei with occasional moderately sized nucleoli; and a zellballen-type architecture with small tumor nests separated by thin fibrous septa containing capillaries.^[5]^ PEComas are generally grossly circumscribed, but some are histologically infiltrative into the surrounding soft tissue. Histologically, PEComas are composed of clear to lightly eosinophilic cells that are arranged into nests, fascicles, and occasionally sheets, often with a radial arrangement around blood vessels. Epithelioid and/or spindle cells (usually both) with a clear to pale eosinophilic cytoplasm are arranged as sheets, nests, and short fascicles, sometimes in a perivascular distribution. There is typically a prominent capillary network. Rare features have included extensive stromal hyalinization and ganglion-like cells. Atypical histological features associated with malignant behavior are noted as follows. An admixture of epithelioid and spindled cells is common. Some cases are predominantly spindled and identical to the so-called clear cell myomelanocytic tumors, and some are predominantly epithelioid and identical to CCST or monotypic epithelioid AML.^[19]^

Most tumors are composed of relatively uniform nuclei of low nuclear grade, but some have higher grade nuclei and prominent nuclear pleomorphism that is identifiable at low magnification. Multinucleated giant cells are common, and, occasionally, giant cells are encountered with a central eosinophilic zone surrounded by a peripheral clear zone reminiscent of the spider cells seen in adult rhabdomyoma.^[32]^ Most tumors have few, if any, mitotic figures (MFs), but some, especially those of higher nuclear grade, may have prominent mitotic activity [greater than 5 MF/50 high-power fields (HPFs)], including atypical MF. Coagulative necrosis and angiolymphatic invasion are uncommonly identified.

In 2005, Folpe et al^[19]^ reported 26 cases of PEComas of soft tissue and gynecologic origin and proposed criteria for the classification of these tumors as “benign,” “of uncertain malignant potential,” and “malignant.” They observed a significant association of tumor size greater than 5 cm, infiltrative growth pattern, high nuclear grade, necrosis, and mitotic activity greater than 1/50 HPF with subsequent aggressive clinical behavior of PEComas. Malignant PEComa seems to be a very aggressive neoplasm leading to multiple metastases and death.^[1,16,19]^ Malignant members of the PEComa family (other than renal epithelioid AML) have also been reported, but firm criteria for malignancy have not yet been established. In 2013, Doyle and Hornick^[33]^ claimed that these factors should be predominant predictors of malignant behavior, such as marked nuclear atypia, diffuse pleomorphism, >2 mitoses per 10 HPF, and metastases. Malignant PEComas typically demonstrate a high proliferative index with Ki-67 immunostaining. This tumor is composed of nests and sheets of usually epithelioid but occasionally spindled cells with a clear to granular cytoplasm and a focal association with blood vessel walls.^[8]^ The histological features predictive of poor outcome in gastrointestinal PEComa are uncertain, but the presence of coagulative tumor necrosis and size greater than 5 cm seem to be associated with early recurrence.^[12]^

In addition to the criteria by Folpe et al,^[19]^ Im et al^[23]^ asserted that large tumor size, high nuclear grade, infiltrative growth pattern, and high proliferative activity seemed to be predictors of aggressive behavior. In addition, Lee et al^[26]^ reported that tumor size, location, and infiltrative growth patterns were considerable prognostic factors. Vascular invasion is uncommon. It is already apparent that a gray zone exists where prognosis cannot be defined with certainty. PEComa with definite evidence of aggressive behavior may be typically suggested by the presence of marked atypia, high mitotic activity, or coagulative necrosis.^[12]^

Knowledge of the patient's clinical history is crucial, but immunohistochemistry is also invaluable. In view of the potential wide differential diagnosis of these tumors, immunohistochemistry is usually required to confirm the diagnosis of PEComa. To date, the precursor lesion or cell of origin has not been identified. However, these tumors characteristically tend to stain positive for HMB-45,^[21]^ which is the most frequently positive melanocytic marker.^[33]^ PEComas typically show immunohistochemical evidence of both smooth muscle and melanocytic differentiation. They show immunoreactivity for both melanocytic (HMB-45 and/or melan-A) and smooth muscle (actin and/or desmin) markers. Defined by the coexpression of melanocytic and muscle markers, PEC tumors do not have predictable histopathological behavior. The therapy consists of radical resection.^[31]^ HMB-45 was the most sensitive melanocytic marker (96% of cases), followed by Melan-A (72%), microphthalmia transcription factor (MiTF) (50%), and desmin (36%).^[19]^ Although uncommon, some cells may also stain positive for pancytokeratins. Nevertheless, more cases should be analyzed to better understand the origin and histogenesis of PEComas.^[1]^

Strong and diffuse expression of CD117 of the PEComas highlights an important differential diagnostic problem between PEComa and GIST because PEComa is a biphasic mesenchymal tumor with GIST-compatible morphology. Because less than 50% of tumor cells are CD117-positive in some cases of GIST, the use of melanocytic markers is mandatory because GISTs are negative for melanocytic markers.^[34]^ Immunohistochemical demonstration of melanocytic differentiation is the most reliable way to distinguish PEComa from GIST.

### 3.4 Treatment and prognosis

PEComa is a rare neoplasm, and no standardized treatment has been established. Currently, surgery is the mainstay of treatment for primary PEComa at presentation, as well as for local recurrences and metastases, with the aim of obtaining clear resection margins. It is possible that PEComas of the colon occurred previously but could not be recognized as such because of the lack of current diagnostic capabilities. Primary excision is usually curative, as most tumors are benign.

For most tumors, surgical resection is the first or only effective treatment. The most effective treatment for gastrointestinal PEComa is surgical resection.^[9]^ Surgical resection of the tumor with the adjacent tissue in the gastrointestinal tract is the mainstay of treatment of the primary tumor and of local recurrence.^[1,11,13,27]^ Tumor size ranges widely, but most are between 4 and 6 cm at the time of excision.^[19]^ In our presented case, metastatic lesions were successfully managed by resection alone. Cheng et al^[17]^ reported a case with a recurrent PEComa of the sigmoid colon with pancreatic metastasis that was treated with surgical resection only. Surgery seems to be the only approach for aggressive cases, as chemo- and radiotherapy have not shown significant positive results.^[1]^

The role of surgical resection and chemo- and radiotherapy is currently not well defined. However, locally advanced or metastatic disease portends a poor prognosis, and strategies incorporating chemotherapy, radiation, and immunotherapy have been reported.^[14,19,34,35]^ Rigby et al^[35]^ reported an 11-year-old girl with metastatic renal PEComa treated with dacarbazine-based chemotherapy and imatinib mesylate. The tumor did not respond to an initial treatment of chemotherapy.

There are obvious difficulties in performing a therapeutic trial mainly due to the rarity of the disease.^[1]^ Park et al^[14]^ first reported a 7-year-old boy treated with surgical resection and adjuvant IFN-α2b immunotherapy for pediatric PEComa of the ascending colon.

Furthermore, potential benefits of adjuvant chemotherapy have not been investigated.^[9]^ Some patients have been treated with adjuvant therapy (including Gleevec), but the efficacy of such therapy is not known.^[35]^ The effect of adjuvant or palliative treatment is uncertain and unpredictable.^[15]^ The outcome of our present case adds to the main evidence base of curative surgery for the treatment of malignant PEComa. Whether the tumor is benign or malignant cannot be predicted, so that the main treatment for the disease may be surgical resection,^[13]^ especially if malignant cases are encountered, as is done with GISTs.^[36]^

Clinically, most PEComas follow a benign course^[7]^; however, malignant PEComas have been increasingly reported over the past 2 decades.^[13–15,17,21,27,34]^ Because relatively few malignant PEComas have been reported, firm criteria for malignancy have not been established. Infiltrative growth or edges, marked hypercellularity, and marked nuclear pleomorphism/atypia may be secondary features suggesting aggressive behavior or malignancy.^[7,33,34,35]^ Most reported malignant PEComas contained necrotic areas, and many revealed high mitotic index.^[1,8,12,15,16,19,37]^ The risk of recurrence and metastasis risk is low if the Ki-67 marker index is less than 1% of PEC tumors.^[34]^ We noted that Ki-67 labeling of 5% of neoplastic cells was observed in PEComas in the colon and rectum that behaved aggressively in Table 1.

In addition, in another report, PEComa was not recognized at initial presentation, and the diagnosis of PEComa was not made until the patient returned with a metastasis.^[34]^ Therefore, metastatic spread of PEComas may, in some cases, be a late complication that presents after many years. This finding highlights both the need for criteria that more accurately predict the behavior of PEComas and the need for long-term follow-up of patients with PEComas, as widespread metastases may present as a late complication.^[34]^

PEComa can appear in many parts of the body, but there have only been a few reports of PEComa of the colon and rectum. Diagnosis is still unclear without a clear definition of malignant PEComa of the colon. Malignant PEComa is an aggressive disease leading to multiple metastases and death, as expected with a high-grade sarcoma.^[1]^ At the 49th postoperative month, we found that liver metastases and metastatic tumor growth progression were slow after first operation. The treatment strategy for malignant PEComa is still ambiguous and controversial, especially in advanced or metastatic disease. It is difficult to diagnose PEComa by extensive imaging studies or tumor markers.

Postoperative patients need to be closely monitored because the treatment protocol for PEComa of the colon and rectum has not reached a worldwide consensus. It appears that long-term follow-up is necessary for all patients with PEComas of the colon because the prognosis of the disease is not entirely known currently.

## 4 Conclusion

To the best of our knowledge, this is the first case of intestinal PEComa of the rectum with liver metastasis without adjuvant chemotherapy or radiotherapy after 2 surgical resections and follow-up for 120 months. At 28 months after segmental hepatectomy, the patient was doing well. There is no gold standard guidance for the diagnosis and follow-up of PEComas. We believe that histopathological studies produce a valid definition for PEComa prognosis and the establishment of a clinical registry for PEComa of the colon seems to be necessary in the future.

## Author contributions

**Conceptualization:** Kung-Hung Lin, Li-Ren Liou.

**Data curation:** Li-Ren Liou.

**Investigation:** Ming-Shan Su.

**Methodology:** Ming-Shan Su.

**Project administration:** Meng-Lin Huang.

**Supervision:** Nai-Jen Chang, Min-Jen Tsao.

**Validation:** Min-Jen Tsao.

**Writing – original draft:** Kung-Hung Lin.
